# Pertussis Epidemic — California, 2014

**Published:** 2014-12-05

**Authors:** Kathleen Winter, Carol Glaser, James Watt, Kathleen Harriman

**Affiliations:** 1 Immunization Branch, California Department of Public Health; 2 Division of Communicable Disease Control, California Department of Public Health

On June 13, 2014, the California Department of Public Health (CDPH) declared that a pertussis epidemic was occurring in the state when reported incidence was more than five times greater than baseline levels. The incidence of pertussis in the United States is cyclical, with peaks every 3–5 years, as the number of susceptible persons in the population increases. The last pertussis epidemic in California occurred in 2010, when approximately 9,000 cases were reported, including 808 hospitalizations and 10 infant deaths, for a statewide incidence of 24.6 cases per 100,000 population (1). During January 1–November 26, 2014, a total of 9,935 cases of pertussis with onset in 2014 were reported to CDPH, for a statewide incidence of 26.0 cases per 100,000. CDPH is working closely with local health departments to prioritize public health activities, with the primary goal of preventing severe cases of pertussis, which typically occurs in infants. All prenatal care providers are being encouraged to provide tetanus, diphtheria, and acellular pertussis vaccine (Tdap) to pregnant women during each pregnancy, ideally at 27–36 weeks’ gestation, as is recommended by the Advisory Committee on Immunization Practices (ACIP) (4), or refer patients to an alternative provider, such as a pharmacy or local public health department, to receive Tdap.

For this analysis, case report forms with preliminary data on demographics, symptoms, clinical course, and exposures were completed by local and state health department investigators through patient interviews and medical record reviews and were available for 8,562 (86%) cases. All cases met either the Council of State and Territorial Epidemiologists definition for confirmed pertussis, its definition for probable pertussis,* or the CDPH definition for suspected pertussis (2).

Disease incidence in California among infants aged <12 months was 174.6 cases per 100,000 during January 1–November 26, 2014, and was significantly higher among Hispanic infants (rate ratio = 1.7; 95% confidence interval [CI] = 1.5–2.1) and lower among Asian/Pacific Islander infants (rate ratio = 0.4; CI = 0.3–0.6) than among white, non-Hispanic infants (Table 1). Of 6,790 cases with available data, 347 patients had been hospitalized, including 275 (79%) who were aged <12 months, of whom 214 (62% of those hospitalized) were aged <4 months. Among hospitalized infants aged <12 months with complete information, 33% required intensive care; few (24%) had received any doses of diphtheria, tetanus, and acellular pertussis vaccine (DTaP) (Table 2). One death was reported in an infant aged 5 weeks at the time of illness onset. Two additional fatal cases in infants who became ill in 2013 were also reported in early 2014; both were aged <5 weeks at the time of illness onset, and one was hospitalized for more than a year before succumbing to pertussis-related complications.

Of 211 (50%) infants aged <4 months whose mothers’ Tdap immunization histories were available, only 35 (17%) had mothers who reported receiving Tdap at 27–36 weeks’ gestation during their most recent pregnancy. Among mothers not vaccinated during pregnancy, 56 (36%) received Tdap within 7 days after delivery.

Disease incidence was also high among older children and adolescents, peaking at 137.8 cases per 100,000 among adolescents aged 15 years (Figure). Among the 2,006 cases in adolescents aged 14–16 years, five patients (0.2%) were hospitalized; four were admitted for ≥2 days, and one was admitted for 5 days. Among the 83% of adolescent cases aged 14–16 years with known vaccination histories, only 2.2% reported never receiving any doses of pertussis-containing vaccine. Of those vaccinated adolescents with complete data, 87% had previously received the Tdap booster vaccine, and the median length of time since prior Tdap dose was 3 years (range = 0–7 years). Of the 1,321 (66%) adolescents aged 14–16 years with known race and ethnicity, rates were highest among non-Hispanic white (166.2 cases per 100,000) adolescents and lower among Hispanic (64.2 per 100,000), Asian/Pacific Islander (43.9 per 100,000) and non-Hispanic black (23.7 per 100,000) adolescents.

## Discussion

Because infants aged <12 months have the greatest risk for hospitalization and death from pertussis, public health strategies have been prioritized towards preventing disease in this age group. During the 2010 pertussis epidemic in California, the main strategy used to protect infants was “cocooning” (i.e., vaccinating contacts of infants so they do not transmit pertussis to the infant). However, this strategy is difficult to implement, and even if all anticipated contacts could be immunized, infants could still be exposed to infected persons in the community.

In 2011, data became available demonstrating efficient transplacental transfer of antipertussis antibodies to the fetus, which might protect vulnerable infants until they are old enough to receive the primary DTaP series beginning at aged 2 months. In that year, ACIP recommended that pregnant women who had never received Tdap receive a dose after 20 weeks’ gestation (3). In 2012, ACIP reviewed data indicating that antipertussis antibody concentrations declined substantially 1 year after vaccination; therefore, ACIP recommended that Tdap be administered during the third trimester of every pregnancy. Since the immune response to Tdap peaks about 2 weeks after administration and the majority of maternal antibodies are acquired by the fetus at 36–40 weeks’ gestation, Tdap is currently recommended at 27–36 weeks gestation to optimize antibody transfer and protection at birth (4,5). Preliminary data indicate that infants born to vaccinated mothers have a lower risk for pertussis early in life (6).

What is already known on this topic?

In the prevaccine and postvaccine eras, pertussis incidence has been cyclical and peaks every 3–5 years. Incidence of reported pertussis has been increasing in the United States since the 1980s despite widespread use of pertussis vaccines. Large outbreaks of pertussis occurred in California in 2010 and in other states during 2011–2012.

What is added by this report?

During January 1–November 26, a total of 9,935 cases of pertussis with onset in 2014 were reported in California, for an incidence of 26.0 cases per 100,000 population. The highest burden of disease is being observed in infants aged <12 months, especially Hispanic infants, and in non-Hispanic white teenagers aged 14–16 years, consistent with the upper age of the cohort of children who have only received acellular pertussis vaccines. Severe and fatal disease continues to occur almost exclusively in infants who are too young (age <2 months) to be vaccinated against pertussis. Few mothers of infants diagnosed with pertussis in California (17%) reported receiving tetanus, diphtheria, and acellular pertussis vaccine (Tdap) during the third trimester of pregnancy, as is recommended by the Advisory Committee on Immunization Practices.

What are the implications for public health practice?

Pertussis incidence is likely to continue to increase in the United States. Prevention efforts should be focused on preventing severe disease and death from pertussis in young infants. The preferred strategy is vaccination of pregnant women during the third trimester of each pregnancy to provide placental transfer of maternal antibodies to the infant. Prenatal care providers are encouraged to provide Tdap to pregnant women (considered best practice) or refer patients to obtain vaccine from an alternative provider, such as a pharmacy or local public health department. Efforts should be made to eliminate barriers to receiving vaccines from prenatal care providers.

Very few mothers of infants with pertussis had received Tdap during pregnancy; many more were vaccinated after delivery, which does not confer any direct protection to the infant and is no longer a preferred strategy. Recently published data indicate that Tdap vaccination coverage among pregnant women was only 19.5% in 2012 across California Vaccine Safety Datalink sites (7). Similarly, in a survey conducted at 100 birthing hospitals in California during October 2013, only 25% of new mothers reported receiving Tdap during pregnancy, whereas an additional 44% received Tdap in the hospital after delivery (CDPH, unpublished data, 2013). However, efforts to increase vaccine coverage have been successful among Northern California Kaiser patients, and in the third quarter of 2014, an estimated 84% of pregnant women received Tdap vaccine in their third trimester (T. Flanagan; Northern California Kaiser; personal communications; November 26, 2014).

Prenatal care providers should vaccinate all pregnant patients with Tdap during the third trimester of each pregnancy, ideally at 27–36 weeks’ gestation, as is recommended by ACIP (4). If Tdap cannot be administered on-site during routine prenatal care visits, CDPH encourages prenatal care providers to take the following steps: 1) provide the patient with a strong recommendation and patient-specific prescription for Tdap; 2) refer the patient to specific alternative sites for vaccine, such as pharmacies, primary care providers, or local health departments; and 3) assess Tdap status at follow-up visits to confirm and record receipt of vaccine. In addition, timely initiation of the primary DTaP infant series is essential for reducing severe disease in young infants. According to the American Academy of Pediatrics, DTaP can be administered to infants at an accelerated schedule, with the first dose administered as early as age 6 weeks, when pertussis is prevalent in the community. Even 1 dose of DTaP might offer some protection against serious pertussis disease in infants (8).

Hispanic infants age <12 months have the highest and Asian/Pacific Islanders of all ages have the lowest rates of disease compared with other racial and ethnic groups. However, the Hispanic overrepresentation among infants disappeared by age 1 year, and disease incidence among older children and adolescents was highest among non-Hispanic whites, similar to trends reported previously in California (1). Nationally, since the 1990s, Hispanic infants have been noted to have higher rates of reported disease and pertussis-related deaths compared with non-Hispanic infants (8). The causes of these disparities are unknown, and data are needed to assess the contributing factors. Current hypotheses attribute the disparities to larger household size and/or cultural practices that increase the number of persons in contact with young infants.

Notably, the peak age of disease incidence beyond infancy increased to age 14–16 years in 2014 compared with the peak among children aged 10 years during the 2010 pertussis epidemic (1). Children and teenagers born in the United State since 1997 have only received acellular pertussis vaccine, and the upper age of this cohort correlates with the peak age in incidence during both epidemic years. Data available since the 2010 epidemic indicate that immunity conferred by acellular vaccines, particularly when used for the primary series, wanes more rapidly than that conferred by older, whole-cell vaccines that were used in the United States from the 1940s to the 1990s. Because of vaccine safety concerns related to whole-cell pertussis vaccines, acellular pertussis vaccines were developed and recommended in 1992 for the 4th and 5th doses of the pertussis vaccine series and for all 5 doses in 1997. Acellular pertussis vaccines are less reactogenic than whole-cell vaccines, but the immunity conferred by them wanes more quickly. Most of the cases among adolescents aged 14–16 years were among those who had previously received Tdap ≥3 years earlier, suggesting that their illness was the result of waning immunity. It is likely that increased incidence will continue to be observed among this cohort in the absence of a new vaccine or more effective vaccination strategy. Although the highest burden of disease is currently being observed in adolescents aged 14–16 years, severe disease is uncommon at this age, and <0.5% of reported cases in this age group resulted in hospitalization. More data are needed to assess the potential benefit and timing of Tdap booster doses.

CDPH is working with local public health departments as well as prenatal and pediatric health care providers, with the primary goal of encouraging vaccination of pregnant women and infants. In addition, CDPH is providing free Tdap to local health departments and community health centers to support vaccination of uninsured and underinsured pregnant women and is working to identify and mitigate barriers to Tdap vaccination for pregnant women. CDPH has been working closely with California local health departments to modify guidance for managing the high burden of disease in older children and teenagers, including school outbreaks of pertussis, by prioritizing follow-up of patients and contacts who are at higher risk for developing severe disease (9).

As long as currently available acellular pertussis vaccines are in use, it is likely that the “new normal” will be higher disease incidence throughout pertussis cycles. The number of reported cases in 2014 has surpassed that of the 2010 epidemic and represents the most cases reported in California in nearly 70 years (1). However, it is important to put the current pertussis epidemic in historical perspective. In the immediate prevaccine era, there were approximately 157 reported cases of pertussis per 100,000 population in the United States, with 1.5 deaths per 1,000 infants (10). Therefore, despite the limitations of currently available pertussis vaccines, they continue to have an important impact on pertussis. Strategies to prevent the most severe cases of pertussis, which occur primarily in young infants, should be prioritized.

## Figures and Tables

**FIGURE:**
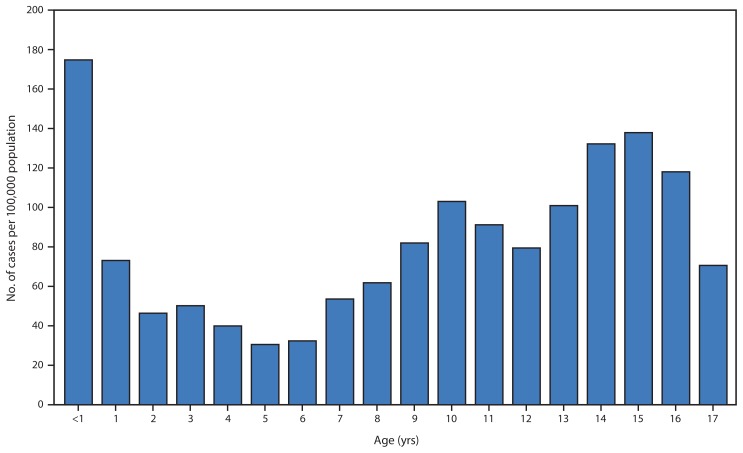
Incidence of pediatric pertussis, by age — California, 2014* * Reported to the California Department of Public Health as of November 26, 2014.

**TABLE 1:** Number and rate of pertussis cases among infants aged <12 months, by race/ethnicity — California, 2014*

Race/Ethnicity	No.	Rate per 100,000	RR	(95% CI)
White, non-Hispanic	169	120.7	Referent	—
Hispanic, all races	551	207.0	1.7	(1.5–2.1)
Black, non-Hispanic	30	110.0	0.9	(0.6–1.4)
Asian/Pacific Islander, non-Hispanic	31	48.5	0.4	(0.3–0.6)
Other/Unknown	132			

**Abbreviations:** RR = rate ratio; CI = confidence interval.

* N = 913. Rates based on population estimates obtained from the California Department of Finance.

**TABLE 2:** Number and percentage of infants aged <12 months hospitalized with pertussis, by selected characteristics — California, 2014*

Characteristic	No.	(%)
**Age group**		
<2 mos	135	(49)
2 mos to <4 mos	79	(29)
4 mos to <6 mos	33	(12)
6 mos to <12 mos	28	(10)
**Vaccination history** †		
DTaP >7 days before onset	53	(24)
No DTaP or <7 days before onset	169	(76)
**Hospital course**		
Median length of stay (days)§	3 (1–50)	
Admitted to intensive care unit¶	71	(33)
Intubated**	18	(8)
Died	1	(1)

**Abbreviation:** DTaP = diphtheria, tetanus, and acellular pertussis vaccine.

* N = 275.

† Out of 222 with known vaccination status.

§ Out of 225 with complete data.

¶ Out of 216 with complete data.

** Out of 237 with complete data.

## Footnotes

* Available at http://wwwn.cdc.gov/nndss/script/casedef.aspx?condyrid=950&datepub=1/1/2014%2012:00:00%20am.
